# Mouth Infections

**Published:** 1916-11

**Authors:** 


### MOUTH INFECTIONS.

Only recently has the part played by the month and its adjacent structures in the production of a great many pathologic processes been demonstrated. The mouth, including the teeth, gums and tonsils, affords a broad surface and readily accessible means of entry for various pathogenic micro-organisms. It is not always essential that visible evidence should be present in the mouth of the focus producing a systemic infection, to prove that the portal of entry was in the mouth. The focus may be discovered only by careful examination with the Roentgen ray. A portal of entry may be present and not directly demonstrable in any way, for example, when the bacteria enter the lymph or blood stream by way of the tonsils. That a focus does exist in the mouth and that it is directly responsible for the pathologic condition has been repeatedly proved by removing the source of infection or by treatment with a proper autogenous vaccine made from bacteria isolated from the pus at the site of the focus. Such treatment often causes the disappearance of the systemic pathologic condition.

Within the mouth there are various channels through which bacteria may enter the system: the tonsils, gums, roots of the teeth, and by way of the esophagus to the stomach and intestine. That the tonsils play an important part in various infections has been demonstrated by many. In a careful bacteriologic and pathologic study of the bacteria removed from the tonsils in various diseases, and injection of these bacteria into animals, Davis[1] was able to reproduce many of these conditions. He studied 113 hypertrophied tonsils from patients with endocarditis, nephritis, arthritis and simple tonsillar cases, isolating the organisms, most of which were streptococci, and injecting them into animals and producing a corresponding condition. The beneficial effects of tonsillectomy, he thinks, furnish the best evidence that the infected tonsils are concerned in the production of arthritic, cardiac, renal and other clinical conditions. Davis[2] again in 1913, in a study of forty-two cases of arthritides, could trace the condition to the diseased tonsils. Capps[3] summarized 1,400 cases of epidemic streptococcic sore throat, and mentions several complications, such as otitis media, erysipelas, arthritis, endocarditis and myocarditis. There were also pleurisy and pneumonia, while peritonitis that occurred resulted in many deaths.

In this connection, the work of Rosenow[4] proved that many ulcers of the stomach are associated with tonsillar infections. He obtained streptococci from some of these ulcers, and after injecting cultures of these organisms into animals, he was able to observe gastric ulcers in them. Stone[5] pointed out the relation between enlarged cervical glands and foci of infection in the tonsils, even though the tonsils may not be hypertrophied or inflamed. The short and direct communication between the tonsils and the lymphatics is responsible for this. In this way there may also result tuberculous adenitis.

That the mouth by way of the esophagus directly offers an entry for bacteria to reach the stomach is evident. The various bacteria are carried in from the dust that gets into the mouth, from the foods, and from infected places in the mouth, particularly the gums. Mayo[6] says that Smithies, in a "microscopic examination of gastric contents from 2,406 different individuals with 'stomach complaint' (dyspepsia, indigestion and the like), showed that irrespective of the degree of acidity of such gastric extracts, bacteria were present in 87 per cent.," and that while the proliferation of the pus-producing organisms, such as the streptococci and staphylococci, were retarded in the gastric juice, bacilli seemed to thrive.

Perhaps equally common as a source of infection are the teeth. The open, exposed, ulcerated or decayed tooth is not always the worst in this respect. More harm may be done by the heavily crowned, capped and bridged teeth, under the poorly fitted margins of which the bacteria flourish and manage either to enter the lymphatics or to send their toxins into the system. There may also be a tiny abscess situated deep down at the root of the tooth. In these cases local manifestations of a focus in the teeth may be entirely lacking, and may be demonstrable only by the Roentgen ray. These so-called "blind abscesses" may remain dormant a long time. Ultimately they open into the mouth by way of the sinus. Often they lead into larger abscesses in the bone, in which toxins are produced, giving rise to septic conditions. Haskin[7] believes that such abscesses, with their subsequent complications, are even more dangerous than the infections resulting from pyorrhea alveolaris. He describes a series of cases among which were complications such as gastric ulcer, anemia and neuritis. It is probable that, depending on the nature of the organism in the abscess, there may result any of the complications so often resulting from tonsillar affections. Rosenow[8] states that these foci are common in patients who for years have suffered from arthritis, neuritis, appendicitis, ulcer of the stomach, goiter, etc., and that persons with perfect health are, as a rule, free from sources of infection in relation to the teeth.

The treatment of the complications secondary to the focus within the mouth consists first of all in removing the mouth infection. Careful examination should be made of the tonsils and teeth, and if the tonsils are found to be hypertrophied or inflamed, even without visible signs of any abscess, they may be removed. Often the abscess may be located deep in one of the crypts. If any visible pus is present, it would be advisable to obtain a culture of the bacteria contained in it. Normal tonsils should not be removed. When the tonsils appear normal, even though there may be no history of tooth involvement, the teeth, nevertheless, should be carefully examined. Poorly fitting crowns should be taken off, and often underneath them may be found the cause of the trouble. Many cases of arthritis have been cured by a correction of the dental work of the mouth, removal or filling of ulcerated areas, and insertion of proper bridge work. Roentgenograms may locate a blind or apical abscess when least suspected. When such has been found, the tooth should be extracted. Often it is possible to drill into the abscess and in this way offer drainage for the pus. In this way also the tooth may be saved. This, however, can best be left to the judgment of the dentist.

Besides the removal of any foci, a mouth wash may be indicated. There are many mouth washes on the market under various trade names. Many of these contain the same ingredients and vary but slightly in their composition from those described in either the Pharmacopeia or the National Formulary. The best mouth washes are those that are alkaline, antiseptic and astringent. Some of the simpler antiseptic and astringent mouth washes are strong solutions of glycerin or of alcohol. Hydrogen peroxide, one part, to three parts of water is a good wash. For ordinary cleansing purposes sodium bicarbonate, in water, will serve the purpose.

Cleanliness of the teeth plays an important part in the asepsis of the mouth. By regular and frequent brushing of the teeth with a good, fairly stiff toothbrush and a simple tooth powder or tooth paste, the accumulation of tartar on and between the teeth may be to some extent prevented. In conjunction with this brushing of the teeth, gargling with a mouth wash will aid in cleaning the mouth. Equally if not more important in the care of the teeth is the periodic visit two or three times a year to a dentist, that tartar may be removed, that caries of the teeth may be early treated, and that the condition of the gums may be noted and pus pockets early discovered.

### PREVENTION.

Prevention of suppuration or other infection in the mouth is of the greatest importance all through life. The following suggestions for preventive measures may be of value:

1. There should be inspection of children's teeth in schools.
2. Every infected area in the mouth must be treated and eradicated if possible as soon as discovered.
3. The public should be taught that a bad tooth or a diseased gum or tonsil is serious, and neglect of such a condition may cause an incurable disease.
4. The mouth of every patient should be examined as part of the physical examination.

5. Roentgenograms of suspected gums or jaws should be taken, and if advisable, a culture from the pus or secretions of the infected region should be made.

6. There should be co-operation of the physician with the dentist to decide on what is best for the correction of mouth defects, whether certain teeth should be filled or pulled, or otherwise treated, and just what is the best treatment for a diseased gum or tonsil. Neither physician nor dentist is infallible, and both should recognize that co-operation is best for the patient.—*Journal A. M. A.*, Nov. 4, 1916.

---

[1] Davis: *Jour. Infect. Dis.*, 1912, x, 148.

[2] Davis, D. J.: Chronic Streptococcus Arthritis, *Journal A. M. A.*, September 6, 1913, p. 724.

[3] Capps, J. A.: Epidemic Streptococcus Sore Throat—Its Symptoms, Origin and Transmission, *Journal A. M. A.*, September 6, 1913, p. 723.

[4] Rosenow, E. C.: The Production of Ulcer of the Stomach by Injection of Streptococci, *Journal A. M. A.*, November 29, 1913, p. 1947.

[5] Stone: *Boston Med. and Surg. Jour*, clxvii, 539.

[6] Mayo, C. H.: Mouth Infection as a Source of Systemic Disease, *Journal A. M. A.*, December 5, 1914, p. 2025.

[7] Haskin: *New York Med. Jour.*, May 16, 1914, p. 979.

[8] Rosenow, E. C.: Mouth Infection as a Source of Systemic Disease, *Journal A. M. A.*, December 5, 1914, p. 2026.

